# MICU1 regulation of mitochondrial Ca^2+^ uptake dictates survival and tissue regeneration

**DOI:** 10.1038/ncomms10955

**Published:** 2016-03-09

**Authors:** Anil Noronha Antony, Melanie Paillard, Cynthia Moffat, Egle Juskeviciute, Jason Correnti, Brad Bolon, Emanuel Rubin, György Csordás, Erin L. Seifert, Jan B. Hoek, György Hajnóczky

**Affiliations:** 1 MitoCare Center, Department of Pathology, Anatomy and Cell Biology, Thomas Jefferson University, Philadelphia, Pennsylvania 19107, USA; 2 Comparative Pathology and Mouse Phenotyping Shared Resource, College of Veterinary Medicine, Ohio State University, Columbus, Ohio 43210, USA

## Abstract

Mitochondrial Ca^2+^ uptake through the recently discovered Mitochondrial Calcium Uniporter (MCU) is controlled by its gatekeeper Mitochondrial Calcium Uptake 1 (MICU1). However, the physiological and pathological role of MICU1 remains unclear. Here we show that MICU1 is vital for adaptation to postnatal life and for tissue repair after injury. MICU1 knockout is perinatally lethal in mice without causing gross anatomical defects. We used liver regeneration after partial hepatectomy as a physiological stress response model. Upon MICU1 loss, early priming is unaffected, but the pro-inflammatory phase does not resolve and liver regeneration fails, with impaired cell cycle entry and extensive necrosis. Ca^2+^ overload-induced mitochondrial permeability transition pore (PTP) opening is accelerated in MICU1-deficient hepatocytes. PTP inhibition prevents necrosis and rescues regeneration. Thus, our study identifies an unanticipated dependence of liver regeneration on MICU1 and highlights the importance of regulating MCU under stress conditions when the risk of Ca^2+^ overload is elevated.

## Abstract (web summary)

Mitochondrial calcium uptake is a highly regulated process, and calcium overload can lead to cell death. Here, using knockout mouse model, the authors show that the mitochondrial calcium uniporter (MCU) regulator MICU1 is needed to prevent calcium overload and promotes survival under liver regeneration and postnatal adaptation-associated stress.

## Graphical abstract

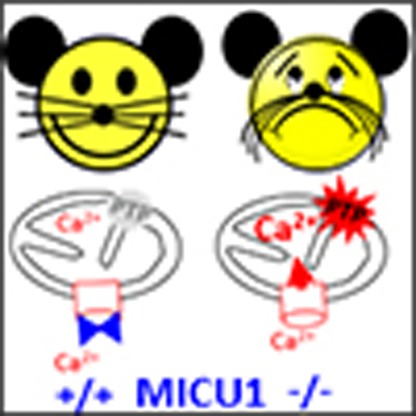

Mitochondrial Ca^2+^ uptake relays cytoplasmic Ca^2+^ signals to the mitochondrial matrix to control vital functions like ATP production but can also overload mitochondria with Ca^2+^ to promote cell death^1^,^2^. Thus, mitochondrial Ca^2+^ uptake needs to be tightly regulated. Ca^2+^ uptake through the outer membrane occurs through the voltage-dependent anion selective channel (VDACs) and across the inner membrane is mediated by a long sought channel, the uniporter^3^,^4^. Recently, essential components of the uniporter have been identified, including the pore-forming subunit, Mitochondrial Calcium Uniporter (MCU)^5^,^6^,^7^ and its Ca^2+^-sensing regulators, MICU1 (refs 8, 9, 10, 11) and MICU2 (refs 12, 13, 14). More precisely, MICU1 has been demonstrated to be a gatekeeper, which is required to close MCU at low cytoplasmic [Ca^2+^] ([Ca^2+^]_c_) and facilitates its activation at high [Ca^2+^]_c_ (ref. 8). Interestingly, a loss-of-function mutation of MICU1 has been recently linked to human disease through alterations in mitochondrial Ca^2+^ handling^15^. Although mitochondrial Ca^2+^ uptake regulation by MICU1 has been well studied in cell culture models^8^,^9^,^12^,^13^, its physiological and pathological role remains unclear.

Previously, MCU was ablated in whole-body knockout mice, which died at around e11.5–13.5 days on a C57BL/6 background and were viable when maintained on a mixed background^16^. In the latter model, and also in cardiac-specific conditional MCU knockout mice^17^,^18^, basal organ functions were maintained and impairments were observed only in the physiological adaptation of skeletal muscle to exercise^16^. Furthermore, the conditional MCU-deficient heart displayed increased resistance to ischaemia-reperfusion injury^17^,^18^. Although, the currently available results do not provide a coherent picture on the physiological relevance of MCU, at least some laboratory mouse strains seem to be able to cope with the loss of mitochondrial Ca^2+^ uptake and show functional impairment only when adaptation is needed to meet an abrupt increase in tissue energy needs. Also, MCU-mediated Ca^2+^ uptake appears to play a pathophysiological role in ischaemia-reperfusion-induced tissue injury. Thus, it is of interest to determine the *in vivo* role of MICU1, which in cultured cells can both suppress and enhance MCU-mediated Ca^2+^ uptake dependent on [Ca^2+^]_c_. In this regard, the liver is an organ of particular interest because [Ca^2+^]_c_ signals are central to stimulation of metabolism as well as for stress responses, and both decoding of [Ca^2+^]_c_ oscillations by mitochondria and protection of mitochondria from calcium overload depend on MICU1 in isolated hepatocytes^8^,^19^.

In this study, we find that whole-body knockout of MICU1 is lethal in the first hours after birth. Indeed, embryos antepartum are at the expected Mendelian ratio but MICU1^−/−^ pups display failure of basic vital functions after birth. To further evaluate the role of MICU1 *in vivo* in a physiological stress response model, liver regeneration following partial hepatectomy (PHx) was studied in mice with a liver-specific MICU1 deletion. Here we show that MICU1 is required for liver regeneration as MICU1 loss leads to an enhanced and sustained pro-inflammatory response post PHx with a failure of hepatocytes to enter the cell cycle and large-scale hepatic necrosis. We demonstrate that Ca^2+^ overload-induced mitochondrial permeability transition pore (PTP) opening is sensitized in MICU1-deficient hepatocytes and that PTP inhibition is sufficient to rescue liver regeneration. Therefore, our data in MICU1-deficient mice reveal that the tight Ca^2+^-dependent control of mitochondrial Ca^2+^ uptake is essential for survival under acute stress conditions and to allow a well-integrated tissue repair response, thus highlighting the critical role of MICU1 in physiological and pathological conditions.

## Results

### MICU1 requirement for transition to *ex utero* life

**Figure 1:**
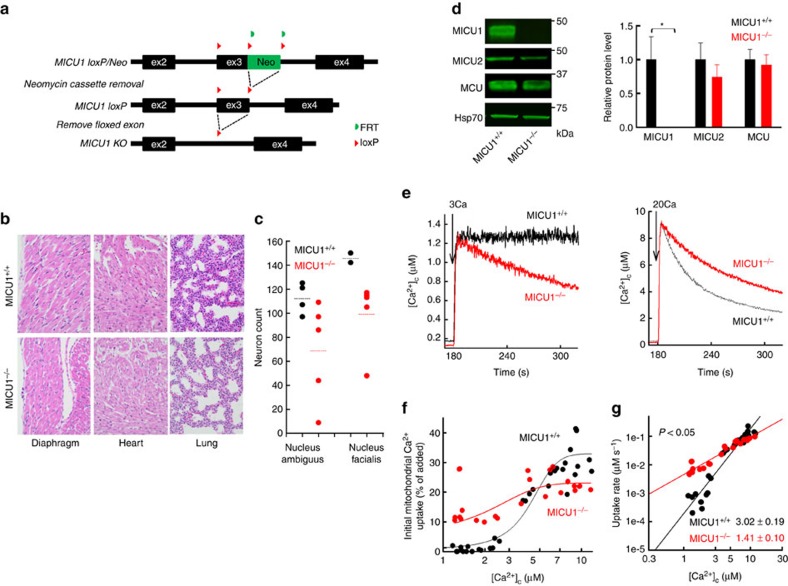
Phenotype of *MICU1*^*−/−*^ mouse and mitochondrial Ca^2+^ uptake in *MICU1*^*−/−*^ MEFs. (**a**) Map of the *MICU1* gene with exon 3 flanked by two loxP sites and a Neomycin selection cassette flanked by flippase recognition target sites (FRTs) and subsequent recombinations for removal of the Neomycin cassette and the exon 3, leading to the truncation of the *MICU1* gene. (**b**) Representative images of haematoxylin and eosin (H&E) staining of diaphragm, heart and lung from *MICU1*^*+/+*^ and *MICU1*^*−/−*^ animals at e18.5 show no morphological abnormalities (× 40). (**c**) Quantification of the neuron count in the nucleus ambiguus and the nucleus facialis in toluidine blue-stained sections of the hindbrain of *MICU1*^*+/+*^ and *MICU1*^*−/−*^ e18.5 animals. Individual points are averages from different animals. Horizontal lines show mean values. Counting of neurons in the nucleus tractus solitarius that contains a subset of inspiratory neurons was not feasible because its borders were less defined. (**d**) Immunoblots of MICU1, MICU2, MCU and Hsp70 in *MICU1*^*+/+*^ and *MICU1*^*−/−*^ MEFs lysates. Relative protein level is displayed in the bar graph; each protein was normalized to Hsp70, and expressed relative to *MICU1*^*+/+*^ MEFs (mean±s.e.m., *n*=4, *P<0.05, Student's *t*-test). (**e**) Representative [Ca^2+^]_c_ time courses of the mitochondrial clearance of a 3 μM or 20 μM CaCl_2_ bolus (3Ca or 20Ca) in permeabilized *MICU1*^*+/+*^ and *MICU1*^*−/−*^ MEFs in the presence of thapsigargin (2 μM) and CGP-37157 (20 μM). (**f**) [Ca^2+^]_c_ dose response of the initial mitochondrial uptake (30 s after CaCl_2_ addition) of different Ca^2+^ boluses recorded as in **e**. The CaCl_2_ doses added were (in μM) 3, 7, 10, 15 and 20 (*n*=4 per group). A sigmoidal fit is displayed for each. (**g**) Double logarithmic plot of the initial rates of Ca^2+^ uptake against the peak [Ca^2+^]_c_. Slope of each linear fit is indicated (mean±s.e.m., *n*=4/group, Student's *t*-test).

MICU1 silencing causes dysregulation of mitochondrial Ca^2+^ uptake in cultured cells^8^,^9^,^10^, and loss-of-function mutations in humans have been linked to neuromuscular disease^15^. The emerging link between MICU1 and human disease emphasizes the need to study MICU1 function *in vivo*^20^. We generated heterozygote MICU1^+/−^ mice utilizing the Cre-loxP system of gene targeting (Fig. 1a). MICU1^+/−^ male and female mice were healthy and fertile. However, crossing these mice did not produce viable MICU1^−*/−*^ pups. Their progeny followed a Mendelian distribution until e18.5, with normal cardiac function by fetal echocardiography (ejection fraction (EF): 79.8±0.7% versus 79.3±1.2%, fractional shortening (FS): 44.5±0.9% versus 45.8±1.1%, MICU1^+/+^ versus MICU1^−/−^, *n*=4, *P*=not significant), but MICU1^−/−^ mice died within hours of birth (Table 1). The few MICU1^−/−^ animals found displayed no gross morphological defects at birth, although several failed a lung float test. Blinded histopathological analysis of the placenta and major organs of e18.5 animals did not reveal structural changes that could have accounted for perinatal lethality (Fig. 1b). To avoid fatalities resulting from labour stress, e18.5 mice were delivered by C-section; all the animals that sustained normal breathing and vocalized intermittently had at least one copy of MICU1. Examination of brainstem nuclei in toluidine blue-stained sections suggested a trend towards decreased numbers of neurons in the nucleus ambiguus (a focus of expiratory control) and the nucleus facialis (Fig. 1c). These observations point to a functional cause of death in MICU1^−/−^ mice, presumably through defective regulation of basic functions postnatally, suggesting a vital role for MICU1 regulation of the MCU during this phase.

We evaluated the impact of MICU1 ablation on mitochondrial Ca^2+^ uptake in primary embryonic fibroblasts generated from MICU1^+/+^ and MICU1^−/−^ embryos. MICU1 ablation was confirmed at mRNA and protein levels, without significant changes in uniporter pore-forming protein MCU or its co-regulator MICU2 (Fig. 1d and Supplementary Fig. 1a). Measurements of the ruthenium red-sensitive clearance of Ca^2+^ added to the cytoplasmic buffer in permeabilized mouse embryonic fibroblasts (MEFs) showed that at low cytoplasmic calcium concentration ([Ca^2+^]_c_), MICU1^−/−^ MEFs took up more Ca^2+^ than MICU1^+/+^ MEFs but displayed a smaller Ca^2+^ clearance at high [Ca^2+^]_c_, at well-maintained inner membrane potential (Fig. 1e and Supplementary Fig. 1b). The dose–response plot (Fig. 1f) showed a lower [Ca^2+^]_c_ threshold and higher Ca^2+^ uptake at low [Ca^2+^]_c_. The double logarithmic plots of initial Ca^2+^ uptake rates against [Ca^2+^]_c_ also showed a lower slope (Fig. 1g), demonstrating decreased cooperativity in activation of mitochondrial Ca^2+^ uptake in MICU1^−/−^ MEFs.

### Acute knockdown (KD) of MICU1 in mouse hepatocytes

**Figure 2:**
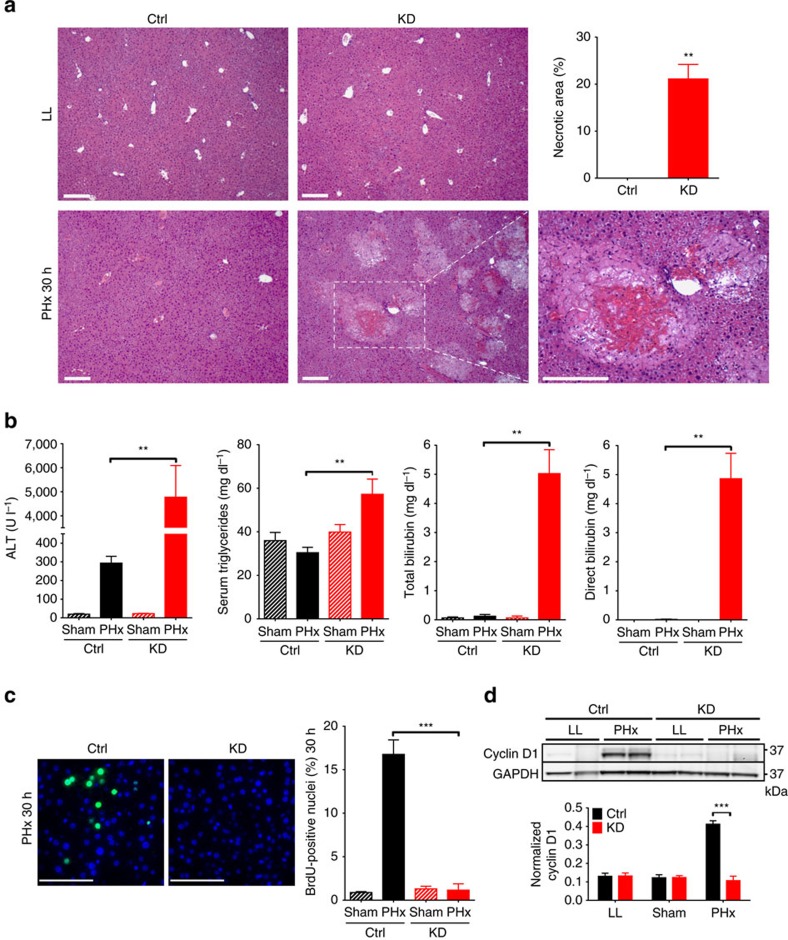
Liver regeneration after partial hepatectomy (PHx) in *MICU1 KD* and Ctrl liver. (**a**) Representative images of haematoxylin and eosin (H&E)-stained liver tissue and quantification of necrotic area in Ctrl and KD mice before (LL, left lateral lobe) and 30 h after PHx (Phx 30 h; collected from the same mouse for individual comparison). Magnified image displays a typical necrotic area in KD liver 30 h after PHx along with quantification. Scale bars, 200 μm (mean±s.e.m., *n*=4 per group). (**b**) Serum levels of alanine aminotransferase (ALT), direct and total bilirubin and triglycerides in Ctrl and KD mice 30 h post PHx, or after sham surgery (mean±s.e.m., *n*=3–6 per group). (**c**) BrdU immunostaining and quantification 30 h post PHx in Ctrl and KD mouse liver. Scale bars, 50 μm (mean±s.e.m., *n*=4 per group). (**d**) Immunoblotting of Cyclin D1 expression and quantification in Ctrl and KD liver lysates before PHx (LL) and 30 h post Sham operated or PHx (mean±s.e.m., *n*=4 per group). Values are mean±s.e.m., **P<0.005, ***P<0.0005 via Mann–Whitney test (**a**) and two-way analysis of variance followed by Tukey's multiple comparison test for the rest.

MICU1 silencing in mouse liver alters hormone-stimulated oxidative metabolism and facilitates mitochondrial Ca^2+^ overload as assessed in isolated hepatocytes^8^. To better understand the physiological role of MICU1, we accomplished a sustained deletion of MICU1 specifically in MICU1^loxP/loxP^ mouse liver through tail-vein injection of AAV8-Cre under a hepatocyte-specific promoter (thyroxine-binding globulin, TBG), leading to hepatocyte-specific MICU1 KD. Three weeks post injection, MICU1 KD was confirmed at both mRNA (98%) and protein level (94%) in isolated hepatocytes and whole liver tissue (Supplementary Figs 2a and 3a). Hepatocyte specificity of MICU1 downregulation was confirmed by no changes in MICU1 mRNA and/or protein level in other organs as well as in the blood (Supplementary Fig. 3b). MCU expression was unchanged, and MICU2 showed a small decrease only in whole liver (Supplementary Figs 2a and 3a). Permeabilized KD hepatocytes showed a lower threshold and decreased cooperativity of Ca^2+^-dependent activation of mitochondrial Ca^2+^ uptake compared with control (Ctrl) cells (AAV8-TBG-Null; Supplementary Fig. 2b–e). Thus, MICU1 KD hepatocytes had a similar mitochondrial Ca^2+^ uptake phenotype as MICU1^−/−^ MEFs and MICU1-silenced HeLa cells^8^,^9^,^12^,^13^, confirming the AAV8-Cre-treated MICU1^loxP/loxP^ mouse as a robust *in vivo* model of acute hepatocyte-specific MICU1 downregulation. Despite alterations in mitochondrial Ca^2+^ uptake in hepatocytes from liver-specific MICU1 KD mice, no gross functional, morphological and/or histological differences were apparent between Ctrl and KD livers (Fig. 2a, LL, Supplementary Fig. 3c and Supplementary Table 1). Gene expression profiling failed to show major differences between KD and Ctrl livers (accession number GSE69801, and Supplementary Fig. 4a,b, LL). Thus, patho-physiological consequences of MICU1 deletion may become apparent only under conditions of acute or chronic stress.

### MICU1 requirement for liver regeneration

We used 70% PHx^21^ as a surgically induced liver-specific stress model^22^. Liver regeneration after PHx is a unique tissue repair response that induces a synchronized replication of differentiated hepatocytes in the remnant liver, while maintaining liver-specific functions^23^,^24^. PHx initiates regeneration through multiple stress signals that induce growth factors and pro-inflammatory cytokines that drive quiescent hepatocytes into the cell cycle to replicate within 30–48 h after surgery, with liver mass recovery within 2–3 weeks (in rodents)^24^,^25^,^26^. Both cytosolic and mitochondrial Ca^2+^ signals may contribute to the onset and early progression of regeneration^27^,^28^,^29^. We hypothesized that aberrant Ca^2+^ signalling in MICU1 KD mice could delay or inhibit regeneration after PHx. In contrast to Ctrl mice, KD mice subjected to PHx were lethargic and unresponsive by 30 h after surgery and at the time when DNA synthesis began in Ctrl mice; their obvious severe distress prevented us from extending the study to later time points for ethical reasons. Histological examination of remnant livers from KD mice showed extensive necrotic foci, with non-necrotic areas of the tissue showing severe microvesicular steatosis, characterized by minute and diffuse fat droplets that do not distort or displace the nucleus (Fig. 2a and Supplementary Fig. 5a). Extensive liver damage and impaired function were evident by dramatically increased serum alanine aminotransferase (ALT) and bilirubin (total and direct). Tissue and serum triglycerides were significantly elevated at 30 h post PHx in KD mice (Fig. 2b and Supplementary Fig. 5b). Hepatocyte proliferation, measured by 5-bromodeoxyuridine (BrdU) incorporation, was significant in Ctrl mice 30 h post PHx, but was completely absent in remnant livers from KD mice (Fig. 2c). This correlated with a total lack of Cyclin D1 expression, 30 h post PHx, in KD liver lysates, compared with its robust expression in Ctrl mice (Fig. 2d). Assessment of the type of cell death suggested a predominant involvement of necrosis rather than apoptosis, as no changes in Bax (Supplementary Fig. 5c) and cytochrome *c* content (Supplementary Fig. 5e) in isolated mitochondria were observed and no cleaved Caspase-3 (Supplementary Fig. 5c) was evident, whereas poly ADP ribose polymerase (PARP) cleavage, a non-specific cell death marker, was increased in KD liver tissue lysate 30 h post PHx (Supplementary Fig. 5d). No differences in tissue histology, serum liver damage markers, Cyclin D1 or other cell cycle markers were detected at 30 h after sham surgery (Fig. 2b–d and Supplementary Fig. 5e). Thus, the absence of MICU1 causes complete failure of liver regeneration, with suppression of hepatocyte proliferation and massive necrosis.

### Sustained pro-inflammatory response post PHx in MICU1-KD

**Figure 3:**
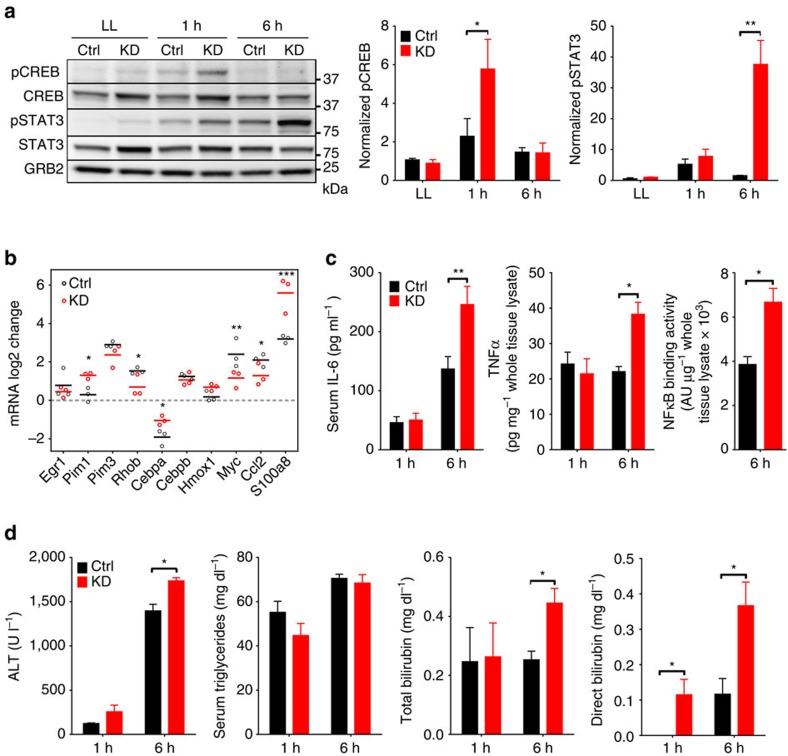
Early response to PHx in *MICU1 KD* and Ctrl liver. (**a**) Immunoblot analysis of phosphorylation level of CREB and STAT3 before (LL) and 1 or 6 h after PHx. Quantification of phospho-STAT3 and phospho-CREB is displayed in bar graphs (mean±s.e.m., *n*=3/group). (**b**) mRNA fold change of genes, relative to LL, 6 h after PHx. Horizontal lines show mean values. (mean±s.e.m., *n*=3/group). (**c**) Serum level of interleukin-6 (IL-6) and tissue level of TNF-α and DNA-binding activity of NF-κB in Ctrl and KD mice 1 and 6 h after PHx (mean±s.e.m., *n*=3–4/group). (**d**) Serum levels of ALT, direct and total bilirubin, and triglycerides in Ctrl and KD mice 1 and 6 h post PHx (mean±s.e.m., *n*=3/group). Values are mean±s.e.m., *P<0.05, **P<0.005, ***P<0.0005 via Mann–Whitney test (**c**-last panel) and two-way analysis of variance followed by Tukey's multiple comparison test for the rest.

To elucidate how MICU1 ablation interfered with hepatocyte proliferation, we evaluated signalling events associated with the early phase of regeneration. These events are characterized by expression of immediate-early genes and activation of stress response signals, followed by release of pro-inflammatory cytokines (tumour-necrosis factor (TNF)-α, interleukin (IL)-6) that drive growth factor production and help transition hepatocytes into the cell cycle^24^,^26^. Remarkably, the early response after PHx was largely unaffected in MICU1 KD mice. Cellular homolog of FBJ murine osteosarcoma viral oncogene (cFOS) and cellular homolog of the viral oncoprotein v-jun (cJUN) upregulation, and JNK activation were not significantly different between Ctrl and KD livers at 1 h (Supplementary Fig. 4c), although a transient hyperactivation/phosphorylation of cAMP response element-binding protein (CREB) in the KD liver was noted at 1 h (Fig. 3a). Several recent studies have reported that phospho-CREB activation downstream of IL-6 or other mediators contributes to the pro-inflammatory environment in the early regenerative response to PHx^30^,^31^. Expression changes of stress-response and proliferation-related genes were not significantly different in Ctrl and KD mice by 1 h post PHx (Supplementary Fig. 4a). However, by 6 h post PHx, downregulation of Rho-b, Myc and Ccl2, and upregulation of Pim1, at levels usually seen after a massive hepatectomy (>80%), and S100a8 were apparent in KD livers relative to Ctrl (Fig. 3b and Supplementary Fig. 4b) and levels of ALT and bilirubin showed early signs of liver damage and functional impairment in KD mice (Fig. 3d). Indeed, some necrotic foci were observed by histology in KD remnant liver as early as 6 h post PHx but not in the Ctrl (Supplementary Fig. 4d). Also, serum IL-6 was elevated in KD mice compared with Ctrl by 6 h post PHx, together with increases in tissue TNF-α, phospho-signal transducer and activator of transcription 3 (STAT3) and nuclear factor-κB activity (Fig. 3a,c), indicating a more elevated pro-inflammatory response in KD mice. Thus, KD mice failed to mount an effective transition to the replication-competent state in response to pro-inflammatory signals after injury, resulting in more sustained inflammation and unresolved tissue damage. A role for mitochondrial regulation of Ca^2+^ homeostasis in resolving the PHx-induced pro-inflammatory state to promote cell cycle entry has not previously been recognized. Can mitochondrial calcium overload account for the defective regeneration in KD mice?

### Rescue of regeneration in MICU1-KD liver by PTP inhibition

**Figure 4:**
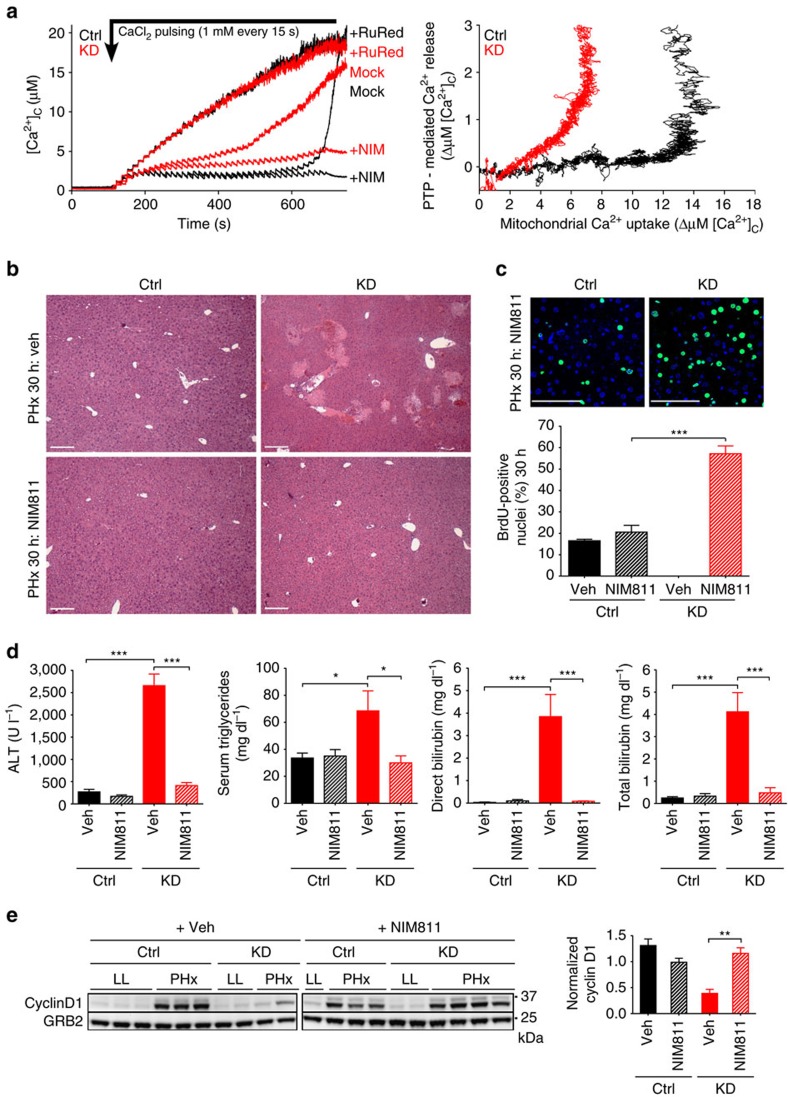
Prevention of mitochondrial Ca^2+^ overload rescues liver regeneration in *MICU1 KD* mice. (**a**) Assessment of Ca^2+^ overload-induced mitochondrial PTP opening in Ctrl and KD permeabilized hepatocytes. Extramitochondrial [Ca^2+^] was recorded during repetitive addition of CaCl_2_ boluses (1 μM each) in the absence (Mock) or presence of RuRed (3 μM) or NIM811 (2 μM). Right panel: Ca^2+^ load dependence of PTP opening shown as Ca^2+^ efflux via PTP (Mock minus NIM811) plotted against the mitochondrial Ca^2+^ uptake (RuRed minus Mock), mean traces of triplicates. Representative of *n*=4 independent experiments. (**b**) Representative images of haematoxylin and eosin (H&E)-stained liver tissue 30 h post PHx showing the absence of necrotic area in both Ctrl and KD liver treated without and with 10 mg kg^−1^ NIM811 (4 separate mice). Liver necrosis was evident in vehicle (Veh)-treated KD mice. Scale bars, 200 μm. (**c**) BrdU immunostaining and quantification 30 h post PHx in Ctrl and KD mouse liver. Scale bars, 50 μm (mean±s.e.m., *n*=5 per group). (**d**) Serum levels of ALT, direct and total bilirubin and triglycerides in Ctrl and KD mice after 30 h of PHx and treated with either vehicle or NIM811. (mean±s.e.m., *n*=5 per group). (**e**) Cyclin D1 expression assessed by immunoblotting in Ctrl and KD liver lysates of Ctrl and KD mice treated with NIM811 or vehicle before (LL) and after 30 h PHx (mean±s.e.m., *n*=3–4/group). Values are mean±s.e.m., *P<0.05, **P<0.005, ***P<0.0005 via two-way analysis of variance followed by Tukey's multiple comparison test.

If MICU1 deletion decreases the threshold for mitochondrial Ca^2+^ uptake, we expect a higher susceptibility to mitochondrial Ca^2+^ overload and increased sensitivity of mitochondrial PTP opening upon [Ca^2+^]_c_ increases^8^. Indeed, we found that permeabilized KD hepatocytes were sensitized to PTP opening (Fig. 4a). Less Ca^2+^ uptake was required to trigger PTP opening in KD than in Ctrl hepatocytes (Fig. 4a, 8.41±1.43 in Ctrl versus 4.58±0.17 ΔμM in KD, *n*=4 independent experiments, *P*<0.05) and this was blocked by the PTP inhibitor NIM811 (Fig. 4a)^32^. To test the role of PTP opening in the regeneration defect and liver damage after PHx in MICU1 KD mice, we treated Ctrl and KD mice with NIM811 (10 mg kg^−1^) or vehicle and assessed its effect 30 h post PHx. Compared with vehicle-treated KD mice, NIM811-treated KD mice were completely protected from liver damage (Fig. 4b) and serum levels of ALT, bilirubin and triglycerides were comparable to Ctrl mice at 30 h post PHx (Fig. 4d). Preventing PTP opening in KD mice by NIM811 was sufficient to rescue cell proliferation, evident as increased BrdU incorporation and upregulation of Cyclin D1 expression (Fig. 4c,e). Interestingly, hepatocyte proliferation was significantly higher in NIM811-treated KD hepatocytes compared with either vehicle or NIM811-treated Ctrl hepatocytes, suggesting accelerated cell proliferation in KD livers when mitochondria are protected from damage (Fig. 4e). Importantly, no differences in the expression level of the NIM811 target cyclophilin D were observed between Ctrl and KD mice (Supplementary Fig. 6a).

Although cyclophilin D is the preferred target of NIM811, cyclophilin A, which plays a role in inflammatory processes *in vivo* can also be affected. We therefore tested whether inhibition of leukocyte migration could contribute to the effect of NIM811, by immunostaining of neutrophils using Ly-6g (Supplementary Fig. 6b). A significant increase in neutrophil count was observed from the time of PHx to 30 h post surgery in Ctrl livers reflecting the previously reported pro-inflammatory component of the response to PHx in mice^33^, and an even larger increase in the MICU1-deficient liver after PHx. In mice treated with NIM811, changes in neutrophil numbers were similar in the Ctrl and decreased in MICU1-deficient tissue. The lack of a NIM811-dependent change in the Ctrl supports the conclusion that NIM811 did not act by suppressing neutrophil migration; the decrease in neutrophil count in the MICU1 KD is presumably secondary to the decrease in hepatocyte death (Supplementary Fig. 6b).

## Discussion

In this study, we have determined the pathophysiological consequences of MICU1 deletion in mouse. Differently from MCU loss^16^, MICU1 deletion validated by genetic, biochemical and Ca^2+^ transport measurements did not interfere with embryonic development on C57BL/6J background. However, every MICU1 KO pup died after birth. Although most of the organs showed normal morphology and histology, several signs, including uninflated lungs, fewer neurons in the brainstem and prolonged gasping indicated that respiratory activity/control might be affected. This phenotype also shares some commonality with the neuronal and muscular impairments displayed by the patients harbouring MICU1 loss-of-function mutations^15^.

The present work and some previous studies by us^8^,^34^ and others^9^,^15^ indicate that loss of MICU1 can impair bioenergetics and cell function both by enhancing mitochondrial Ca^2+^ uptake at resting [Ca^2+^]_c_ or during prolonged [Ca^2+^]_c_ elevations, and by decreasing mitochondrial Ca^2+^ uptake during [Ca^2+^]_c_ spikes and oscillations. Several lines of evidence link mitochondrial Ca^2+^ overload to neuronal dysfunction^35^,^36^, and this study shows that upon hepatocyte-specific MICU1 deletion, the liver failure caused by tissue injury is mediated by mitochondrial Ca^2+^ overload. For these reasons, we propose that the impaired adaptation to extra-uterine life is likely due to altered mitochondrial Ca^2+^ handling with an overload component. The recent observations on the MCU-deficient mouse phenotype and the present findings on the MICU1^−/−^ mice converge to support the notion that increased Ca^2+^ flux through the uniporter fuels an important pathogenic pathway.

The dramatic failure of liver repair after PHx in the MICU1-deficient mice demonstrates this pathogenic potential of deregulated mitochondrial Ca^2+^ and points to an unexpected sensitivity of the regenerating liver to calcium overload. Despite a large number of gene ablation studies in the context of liver regeneration it is unusual for these to result in massive liver necrosis as reported here, as liver regeneration can often be compensated by eliciting the differentiation and proliferation of progenitor cells. Although previous studies had identified a role for Ca^2+^ signalling in the early response to PHx^27^,^28^, the underlying mechanism has remained poorly characterized. In the present study, the initial priming response to PHx was largely unaffected by the MICU1 defect in hepatocytes, but the onset of the cell cycle appeared to be suppressed, coinciding with evidence of a persistent pro-inflammatory state with the onset of patchy necrosis at 6 h after surgery. We were unable to detect significant changes in the Ca^2+^ content of mitochondria isolated from the remnant liver, presumably due to a lack of Ca^2+^ retention during the isolation procedure. However, the complete protection against PHx-induced liver failure in MICU1 KD mice afforded by NIM811 treatment clearly points to PTP opening because of mitochondrial calcium overload as the cause of tissue injury.

It is interesting to note the observation of enhanced hepatocyte proliferation at 30 h after PHx in NIM811-treated MICU1 KD mice. Possible reasons for this effect remain to be established and our data do not allow us to assess whether it is due to enhanced mitochondrial functions under conditions of calcium overload or to changes in cytosolic or nuclear Ca^2+^ signalling processes that drive regeneration. Loss of MICU1 could lead to a suppressed [Ca^2+^]_c_ response when mitochondria are protected from calcium overload, with potentially decreased activation of calcineurin, resulting in an enhanced or more sustained increase in protein phosphorylation in the early phase of regeneration. We cannot exclude the possibility that NIM811 may act on other targets that affect cell proliferation. However, NIM811 has been shown not to exert inhibitory effects on calcineurin^32^.

**Figure 5:**
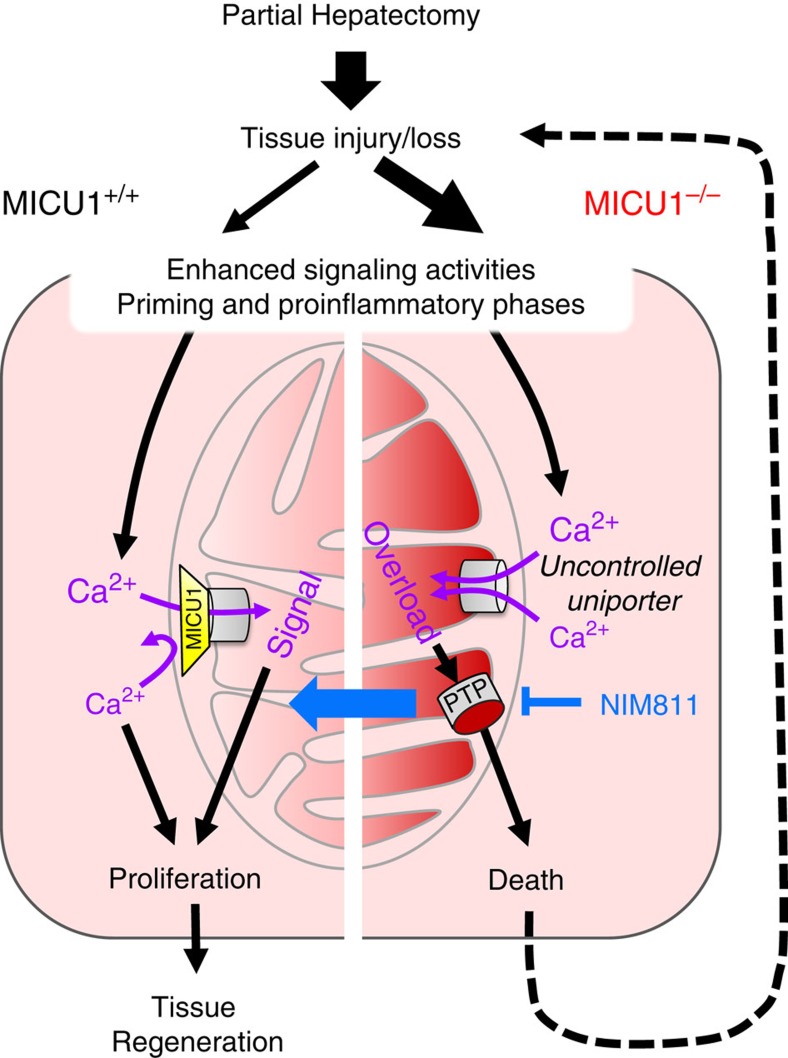
Loss of control over mitochondrial Ca^2+^ uptake turns regeneration signals to death signals in MICU1-deficient hepatocytes. Partial hepatectomy, a surgical model for extensive tissue injury/loss in the liver, reprogrammes the fully differentiated hepatocytes in the remnant liver to proliferate in order to regenerate liver parenchyma. Reprogramming involves the synchronized priming of hepatocytes by growth factors and pro-inflammatory cytokines to obtain replicative competence. These mediators engage a broad range of intracellular signals, including Ca^2+^, which in addition to driving cytosolic effectors, can propagate to the mitochondrial matrix and control metabolic enzyme activities to support the proliferative response. Mitochondrial Ca^2+^ uptake is mediated by the Ca^2+^-gated mitochondrial Ca^2+^ uniporter complex. Ca^2+^ gating occurs through MICU1 (yellow trapezoid), which associates with the MCU pore (grey cylinder) from the intermembrane space side to set the threshold for Ca^2+^ activation. As a result, mitochondrial Ca^2+^ uptake occurs largely through local, high [Ca^2+^] elevations, whereas smaller events and global Ca^2+^ fluctuations are repelled (left-side purple arrows). Upon loss of MICU1 control over the Ca^2+^ uptake threshold, smaller [Ca^2+^] elevations can activate uniporter-mediated uptake (right-side purple arrows). Ca^2+^ signalling events occurring after PHx can thereby lead to mitochondrial Ca^2+^ overload to enhance permeability transition pore (PTP) opening and switch the cell's fate from proliferation to a death track. Large-scale hepatocyte death will overwhelm the functional capacity of the remaining liver cells resulting in organ failure (dashed arrow). Pharmacological inhibition of the PTP by NIM811 rescues mitochondrial competence to support proliferation even under the higher Ca^2+^ load in MICU1-ablated cells (thick blue arrow), which may provide the stimulus for accelerated cell proliferation under these conditions.

In summary, we show that MICU1 helps maintain mitochondrial Ca^2+^ homeostasis in liver and other tissues. Following PHx-induced surgical stress, MICU1 is required for protection against mitochondrial calcium overload in order to allow liver regeneration to proceed. In the absence of MICU1, Ca^2+^-dependent activation of the PTP occurs and the ensuing cell death would augment the pro-inflammatory phase and impair this transition (see schematic in Fig. 5). Specifically, how elevation of cytosolic Ca^2+^ promotes the transition of primed hepatocytes to the proliferative state and whether mitochondrial Ca^2+^ uptake itself is required for this transition or serves a bystander role is open to debate. MCU deletion in mice either was embryonic lethal or showed a mild muscle phenotype dependent on the genetic background^16^,^37^. Thus, the physiological relevance of mitochondrial Ca^2+^ uptake needs to be further tested. Our study of the MICU1-deficient mice reveals that the tight Ca^2+^-dependent regulation of mitochondrial Ca^2+^ uptake is essential for survival under acute stress conditions and to allow a well-integrated tissue repair response. Thus, alterations in MICU1 activity can play a critical role in pathological conditions where mitochondrial control of cellular Ca^2+^ homeostasis is required.

## Methods

### Generation of the MICU1^KO/KO^ mice

All animals were used in accordance with mandated standards of humane care and were approved by the Thomas Jefferson University Institutional Animal Care and Use Committee. To obtain MICU1 knockout mice, the Cre-loxP system was used to target exon 3 of the *MICU1* gene for removal. *MICU1*^*WT/loxP*^*FLP*^*+/0*^ mice (on C57BL/6J background) were generated by Ingenious Targeting Laboratory, then delivered to the Thomas Jefferson University for subsequent breeding. *MICU1*^*WT/loxP*^*FLP*^*+/0*^ were bred with wild-type C57BL/6J mice to remove the FLP transgene. A successive generation of breeding was undergone to generate *MICU1*^*loxP/loxP*^ mice, which were then bred with germline-expressing Cre-eIIa mice (Jackson Laboratories; C57BL/6J background). These offspring were bred with homozygous floxed mice to remove the Cre transgene, and establish whole-body heterozygous knockout mice *MICU1*^*KO/loxP*^(referred to as *MICU1*^*+/−*^). Heterozygote male and female mice were bred to attempt to generate whole-body knockouts (*MICU1*^*−/−*^) as well as control homozygous floxed littermates (*MICU1*^*+/+*^). Genotype of mice was determined using the following primer pairs: 1—5′-GGTGGAGTCAAAGGGAGGAACAG-3′, 5′-GCCCCATCTATGATAATGTTAAGC-3′ (loxP: 519 bp, wt: 341 bp); 2—5′-ATGAGCATGAAGTGATGACCCGAC-3′, 5′-GCCCCATCTATGATAATGTTAAGC-3′ (loxP: 1.5 kb, ko: 500 bp). Only male mice were used for experiments.

### Histological analysis of the embryos

Necropsy was performed on post-natal mice found within breeding cages: lung float test, weight and size measurements, visual examination of major organs, as well as examination of palate formation were performed. E18.5 mice delivered by C-section were stimulated to breath and closely monitored for signs of sustained breathing as well as vocalization. Mice displaying vitality were then successfully fostered with host mothers.

For histological analyses, *MICU1*^*+/+*^ (*n*=5) and *MICU1*^*−/−*^ (*n*=6) e18.5 fetuses were decapitated after C-section, fixed with their placenta by immersion in Bouin's solution for 48 h and then transferred to 70% ethanol. All specimens were processed routinely into paraffin and sectioned at 5 μm at the Comparative Pathology and Mouse Phenotyping Shared Resource (Ohio State University). Sections were stained with haematoxylin and eosin. Tissue trimming and sectioning depended on the nature of the specimen. The head was trimmed into five coronal (transverse) levels: rostral nose, caudal nose (through eyes), forebrain (targeting the hippocampus and diencephalon), midbrain and hindbrain. The abdomen was bisected in the longitudinal plane just to the left of the mid-line, and three-step sections were taken from each half at 300 μm intervals (yielding three sections per animal). Placenta was cut in half, and both halves were processed. A coded (‘blinded') histopathological paradigm was done to examine ∼40–45 major organs and tissues for abnormalities. Lesions were scored using a tiered, semi-quantitative scale: within normal limits, or minimal, mild, moderate or marked changes. After hindbrain sections were evaluated initially, 4-μm-thick step sections were acquired every 40 μm until 30-step sections had been harvested. The sectioning protocol was designed to evaluate major brainstem nuclei associated with control of respiration and facial movements, which have been linked to early neonatal death^38^. The positions of all three nuclei were approximated using landmarks defined in a well-recognized neuroanatomic atlas^39^. The locations of the main inspiratory centre (that is, nucleus of the solitary tract) and expiratory centre (that is, nucleus ambiguus) were defined as present or not based on regional cytoarchitecture and the tinctorial characteristics of the neurons. The boundaries of the solitary tract nucleus could not be defined with certainty, so no counts were gathered from this structure. The large neurons in the nucleus ambiguus (considering the compact, subcompact and loose parts collectively) as well as the facial nucleus could be identified and were counted in some animals. However, the initial facing of the blocks partially or completely effaced these latter two nuclei in some animals, thereby preventing collection of reliable cell counts from them.

### Generation of the liver-specific MICU1 knockdown mice

To generate liver-specific *MICU1 knockdown (KD)* mice, *MICU1*^*loxP/loxP*^ male mice between 10 and 12 weeks were injected via the tail vein with an AAV8-Cre under the control of a hepatocyte-specific promoter (TBG; 1.3 × 10^11^ plaque-forming units per mouse; Penn Vector Core). AAV8-TBG-Null-injected littermates were used as controls. Animals were used for experiments 3–5 weeks post injection.

### Cell culture

MEFs were isolated from e14.5 embryos by trypsin digestion and then immortalized. MEFs were cultured in DMEM (ATCC 30-2002) supplemented with penicillin, streptomycin at 37 °C/5% O_2_. Primary hepatocytes were isolated by *in situ* retrograde perfusion with collagenase (Sigma) as previously described^8^. Only preparations with >90% viability were used for subsequent experiments.

### Mitochondrial Ca^2+^ uptake and membrane potential

Fluorometric measurements of mitochondrial [Ca^2+^]_m_, cytosolic [Ca^2+^]_c_ and ΔΨ_m_ were performed as previously described^8^. Briefly, saponin-permeabilized hepatocytes (2 millions) or MEFs (2.4 mg) were resuspended in 1.5 ml of intracellular medium containing 120 mM KCl, 10 mM NaCl, 1 mM KH_2_PO_4_, 20 mM Tris-HEPES at pH 7.2, and supplemented with proteases inhibitors (leupetin, antipain, pepstatin, 1 mg ml^−1^ each), 2 mM MgATP, 2 μM thapsigargin (Enzo) and maintained in a stirred thermostated cuvette at 35 °C. Assays were performed in the presence of 20 μM CGP-37157 (Enzo) and 1 mM succinate using a multiwavelength-excitation dual-wavelength-emission fluorimeter (DeltaRAM, PTI). The extramitochondrial Ca^2+^ concentration [Ca^2+^]_c_ was assessed using the ratiometric Ca^2+^ probe Fura2-FA (1.5 μM, Teflabs) or Fura-loAff (formerly Fura-FF; 1 μM, Teflabs). Δψm was measured with 1.5 μM TMRM (Invitrogen). Fura and TMRM fluorescence were recorded simultaneously using 340–380 nm excitation and 500 nm emission, and 545 nm excitation and 580 nm emission, respectively. Complete depolarization (maximum de-quench of TMRM fluorescence) was elicited using of the protonophore FCCP (2 μM). Calibration of the Fura signal was carried out at the end of each measurement, adding 1 mM CaCl_2_, followed by 10 mM EGTA/Tris, pH 8.5.

### Animal surgery

The liver-specific *MICU1 KD* male mice between 13 and 16 weeks old (3 weeks post AAV8 injection) underwent 70% PHx based on the surgical methods outlined by Mitchell and Willenbring^40^. Briefly, the animals were anaesthetized with isoflurane, a midline incision was made followed by a segmental ligation and excision of the left-lateral and medial lobes of the liver. The sham surgery involved the gentle manipulation of the liver lobes without the removal of liver tissue. The left-lateral lobe was immediately (within 10 s) freeze clamped using liquid nitrogen cooled aluminum clamps, as described^41^, to prevent post-mortem changes in metabolites and protein phosphorylation. The medial lobe was fixed in 10% neutral buffered formalin (NBF). The abdominal cavity was rinsed with warm lactated Ringer's solution, the abdominal muscle layer sutured and the skin was closed with wound clips. Following surgery, the animals were given subcutaneous lactated Ringer's solution (1 ml per animal) and buprenorphine (0.1 mg kg^−1^) and placed in a fresh cage under a heat lamp with *ad libitum* access to hydrogel (Contact ClearH_2_O) and food. For the PTP inhibition studies, mice were administered NIM811 (10 mg kg^−1^) 30 min before PHx surgery. 50 mg of NIM811 (Norvartis) was dissolved in 1 ml of dimethylsulphoxide/Cremophor EL (3:7) mixture. The stock was 50-fold diluted in 0.9% saline before administering it to the animals.

Animals were given intraperitoneal injections of BrdU solution (Sigma; 150 mg kg^−1^) in 0.9% sterile saline 2 h prior to sacrifice. At specified times after PHx, animals were anaesthetized with isoflurane, blood was collected from the vena cava, then the remnant right-superior lobe and the right-inferior lobe were removed and, respectively, freeze clamped or fixed in 10% NBF. Collected blood was incubated at room temperature for 30 min, centrifuged and serum was collected and then flash frozen for further analysis.

### Histological and immunofluorescence analysis

10% NBF fixed samples were paraffin-embedded, sectioned and stained for hematoxylin and eosin by the Kimmel Cancer Center pathology core facility (Thomas Jefferson University) and evaluated by Dr. Rubin, an expert hepatopathologist. BrdU immunostaining was performed using Anti-BrdU antibody (AbD Serotec, 1:100), which was detected using Anti-Mouse Alexa Fluor-488 secondary antibody (Life technologies). For neutrophil identification, sections were immunostained using Anti-Ly6G (BD Pharmingen, 551459, 1:400), which was detected with Anti-Mouse-HRP (ThermoFisher Scientific), counterstained with haematoxylin, rehydrated and mounted. Quantification of BrdU incorporation was performed using ‘CellProfiler cell image analysis software' and neutrophil count and necrotic foci were quantified using ‘ImageJ software'. For quantification, 15 × 20 fields were scored per animal.

### Biochemical analysis

For western blotting, freeze-clamped tissue was homogenized in lysis buffer containing 25 mM HEPES (pH 7.4), 150 mM NaCl, 2.5 mM EGTA, 10% glycerol, 0.5% deoxycholic acid (DOC), 1% IGEPAL, 0.1% SDS, 1% Triton X-100, 2 mM sodium floride, 2 mM imidazole, 1.15 mM sodium molybdate, 2 mM activated sodium orthovanadate, 4 mM sodium tartrate dihydrate, 1 mM sodium pyrophosphate, 1 mM β-glycerophosphate and cOmplete protease inhibitor cocktail (Roche Diagnostics, Basel, Switzerland). Cell lysates were prepared in RIPA buffer supplemented with proteases inhibitors (leupetin, antipain, pepstatin, phenylmethyl sulphonyl fluoride, Sigma). Proteins were analysed by western blotting after separation by SDS–PAGE on 10% TGX gels (Bio-Rad) or 4–12% Bis-Tris NuPAGE gradient gels (Life Technologies). The following antibodies were used for primary overnight incubation: MICU2 (Abcam (Ab)—ab101465, 1:500), Cyclophilin D (Ab—ab110324, 1:1,000), MICU1 (Sigma (Sg)—HPA037480, 1:400), Cytochrome c (BD Pharmingen—556432, 1:500), MCU (Sg—HPA037480, 1:500); CyclinD1 (Thermo Scientific (TS)—MA1-39546, 1:50), Hsp70 (TS—MA1-91159, 1:1,000); Bax (Cell Signaling Technology (CST)—14796, 1:1,000), cFOS (CST—4384, 1:1,000), cJUN (CST—9165, 1:1,000), p cJUN (Ser73) (CST—3270, 1:1,000), Caspase—3 (CST—9662, 1:1,000), CREB (CST—9197, 1:1,000), pCREB (CST—9198, 1:1,000), JNK54 (CST—9252, 1:1,000), p JNK54 (Thr185/Tyr185) (CST—9251, 1:1,000), PARP (CST—9532, 1:1,000), STAT3 (CST—4904, 1:1,000), p STAT3, (CST—9145, 1:1,000), GRB2 (Santa Cruz Biotechnology—255, 1:1,000), GAPDH (Millipore—MAB374, 1:1,000). Membranes were scanned on the Odyssey scanner (Licor) or Kodak Image Station 440CF. Western blot densitometries were performed using ImageJ (NIH) or Image Studio lite software and normalized to levels of GRB2, GAPDH or Hsp70. Uncropped scans of the most important western blots are shown in Supplementary Fig. 7.

ELISA kits were used to measure serum levels of IL-6 (eBiosciences) and TNF-α (eBiosciences) according to the manufacturer's instructions. Tissue triglyceride was extracted with ethanolic KOH as described^42^. Triglycerides (serum and tissue) were measured using Stanbio Triglyceride Liquicolor reagent (Stanbio) as per the manufacturer's instructions. Nuclear factor-κB DNA-binding activity in 100 μg of total tissue lysate was measured using the transcription factor assay from Cayman Chemical. Serum direct bilirubin and total bilirubin were measured using the bilirubin assay kit (Sigma).

### RNA isolation and quantitative PCR (qPCR)

Total RNA was isolated from frozen liver samples (∼15 mg) using Animal Tissue RNA Purification Kit (Norgen Biotek) and from isolated hepatocytes or MEFs using TRIzol reagent (Ambion). RNA concentration was measured by ND-1000 (NanoDrop). cDNA was synthesized using SuperScript III (Invitrogen) and used for qPCR reactions using SYBR Green (Invitrogen) on an ABI Prism 7000 sequence detection system (Life Technologies). Data were analysed using the comparative ΔΔ*C*_t_ method. *C*_t_ of the gene of interest was normalized to that of β-actin. Primers used are: Mice MICU1 (F-5′-AACAGCAAGAAGCCTGACAC-3′, R-5′-CTCATTGGGCGTTATGGAG-3′), Mice MICU2 (F-5′-GGAGCGTAAAACACTGGTC-3′, R-5′-GTAAGCAAGAAAAGATACTCGG-3′), Mice MCU (F-5′-TACTCACCAGATGGCGTTC-3′, R-5′-GTCCTCTAACCTCTCCAC-3′), β-actin (F-5′-CAACACCCCAGCCATG-3′, R-5′-GTCACGCACGATTTCCC-3′).

### High-throughput qPCR

High-throughput gene expression analysis was performed following standard BioMark (Fluidigm) qPCR protocol #8. cDNA was synthesized from total RNA (1.2 μg) using EasyScript Plus cDNA Synthesis Kit (Applied Biological Materials) and stored at −20 °C. Primers were designed using Universal Probe Library Assay Design Center (Roche Applied Science, https://www.roche-applied-science.com/sis/rtpcr/upl/index.jsp). The primers and probes for those assays that passed the quality control test and were used in this study are listed in Supplementary Table 1. cDNA (100 ng, calculated from initial RNA) samples were pre-amplified for 12 cycles using TaqMan PreAmp Master Mix (Applied Biosystems). qPCR reactions were performed using BioMark Dynamic Arrays (Fluidigm) with 40 cycles of amplification (15 s at 95 °C, 5 s at 70 °C, and 60 s at 60 °C). *C*_t_ values were calculated by the Real-Time PCR Analysis Software (Fluidigm) and software-designated failed reactions were discarded from analysis. Relative gene expression was determined by the ΔΔ*C*_t_ method. Modified geNorm algorithm in R was used to identify the set of most stable reference genes, *Idh3B* and *Mrpl16* were selected for normalization.

### Microarray gene expression analysis

Global gene expression analysis was performed using the Affymetrix Mouse Gene 2.0 ST arrays following the standard manufacturer's protocol. The raw expression data were log-transformed and normalized across all the samples using the Robust Multiarray Averaging algorithm. Normalized data are deposited in GEO database (accession number GSE69801).

### Statistical analysis

Data are expressed as mean±s.e.m. Experiments were performed at least three times, in duplicates or more. Statistical analysis was performed using the Student's *t*-test or Mann–Whitney test for comparison between two groups and ANOVA-2 for others. (*P<0.05, **P<0.005, ***P<0.0005.)

## Additional information

**Accession codes:** Microarray-based gene expression data have been deposited with the GEO database with accession number GSE69801.

**How to cite this article:** Antony, A. N. *et al.* MICU1 regulation of mitochondrial Ca^2+^ uptake dictates survival and tissue regeneration. *Nat. Commun.* 7:10955 doi: 10.1038/ncomms10955 (2016).

## Supplementary Material

Supplementary Information Supplementary Figures 1-7 and Supplementary Table 1

## Figures and Tables

**Table 1:** *MICU1* ^*−/−*^ mice showing Mendelian distribution until e18.5 and death after birth.

**Age**	**Total # embryos**	**# Litters**	**# Animals observed (expected)**				**# (%) Dead pups**		
			**+/+**	**+/−**	**−/−**	Unclear*	**+/+**	**+/−**	**−/−**
e14.5	48	6	9 (12)	25 (24)	12 (12)	2	—	—	—
e18.5	76	9	23 (19)	32 (38)	18 (19)	3	—	—	—
P0	188	38	53 (47)	110 (94)	14 (47)	11	11 (15)	13 (11)	14 (100)

Numbers (#) of observed and expected (in brackets) animals after C-section at embryonic days e14.5 and e18.5, and at birth (P0) are indicated on the left side of the table. Postnatal lethality is shown on the right side with numbers and percentage (in brackets) of dead pups observed at P0.

^*^Resorption or disappearance precluded any genotyping.
