# Supplementary material for: MICU1 regulation of mitochondrial Ca2+ uptake dictates survival and tissue regeneration
Source: Nat Commun. 2016 Mar 9;7:10955. doi: 10.1038/ncomms10955 (PMC4786880; doi:10.1038/ncomms10955)
Supplement: Supplementary Information — Supplementary Figures 1-7 and Supplementary Table 1 [file ncomms10955-s1.pdf]

Supplementary Figure 1.

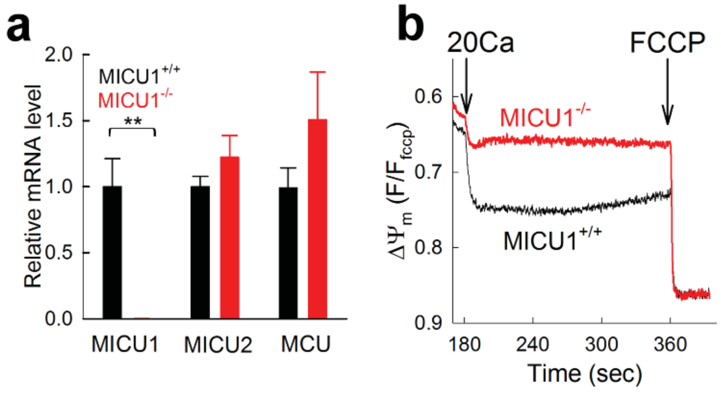

(a) mRNA level of MICU1, MICU2 and MCU in $MICU1^{+/+}$ and $MICU1^{-/-}$ MEFs. Relative mRNA levels are reported using β-Actin as an endogenous control and normalized to the $MICU1^{+/+}$ MEFs for each gene (mean ± SEM, n=5-6, **$p<0.005$, Student's t test). **(b)** Representative recordings of $\Delta\Psi_m$ with TMRM in $MICU1^{+/+}$ and $MICU1^{-/-}$ MEFs before and after the addition of 20 μM $CaCl_2$. The measurement was done simultaneously with the measurement of $[Ca^{2+}]_c$ shown in Figure 1F, right.

Supplementary Figure 2.

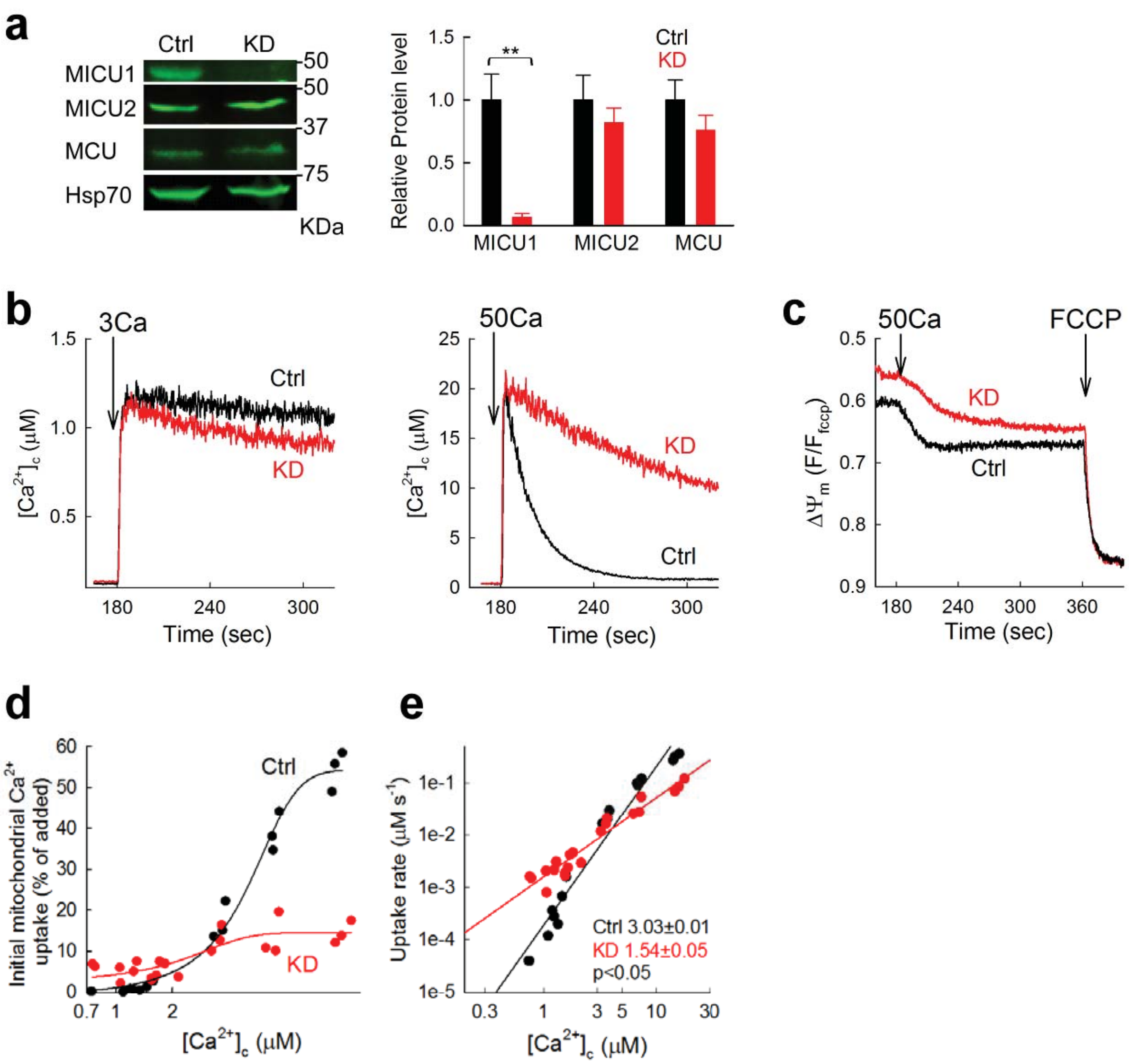

(a) Representative immunoblotting of MICU1, MICU2, MCU and Hsp70 in primary mouse hepatocytes isolated from $MICU1^{loxP/loxP}$ mice three weeks after tail-vein injection of liver-specific AAV8-Null (Ctrl) or AAV8-Cre (KD). Relative protein level is displayed in the bar graph. Each protein was normalized to Hsp70, and normalized to Ctrl hepatocytes (mean ± SEM, n=4, **$p<0.005$, Student's t test). (**b)** Representative time courses of the mitochondrial clearance of the $[Ca^{2+}]_c$ rise upon addition of a 3 μM or 50 μM $CaCl_2$ bolus (3Ca or 50Ca) in permeabilized

Ctrl and KD hepatocytes in the presence of thapsigargin (2 μM) and CGP-37157 (20 μM). **(c)** Representative recordings of $\Delta\Psi_m$ with TMRM in Ctrl and KD hepatocytes before and after the addition of 50 μM $CaCl_2$, as shown in Supplementary Figure 1b. **(d)** $[Ca^{2+}]_c$ dose response of the initial mitochondrial uptake (30s after $CaCl_2$ addition) of different Ca^{2+} boluses in permeabilized Ctrl and KD hepatocytes. The $CaCl_2$ doses added were (in μM) 3, 5, 7, 10, 20 and 50 (n=4/group). A sigmoidal fit is displayed for each. **(e)** Double logarithmic plot of the initial rates of Ca^{2+} uptake against the peak $[Ca^{2+}]_c$. Slope of each linear fit is indicated (mean ± SEM, n=2-4, Student's t test).

Supplementary Figure 3.

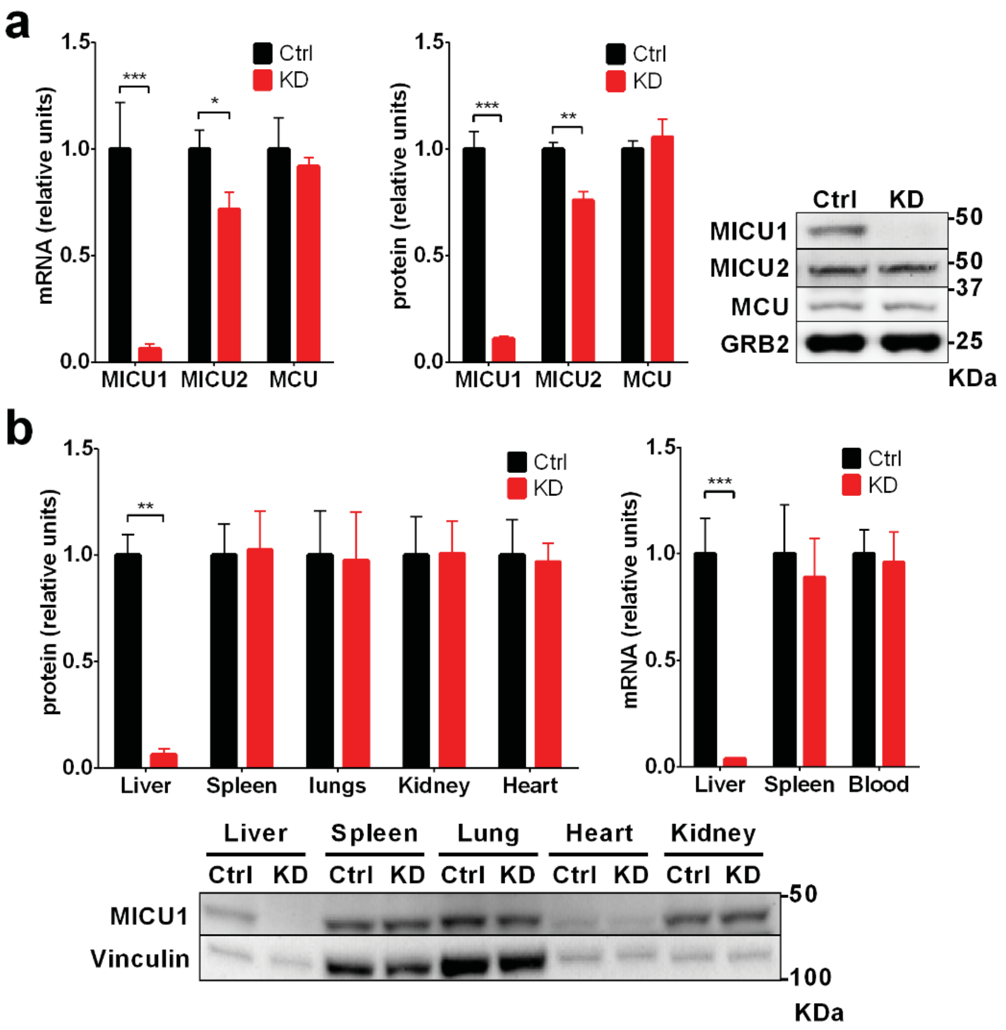

Supplementary Figure 3. (Cont'd)

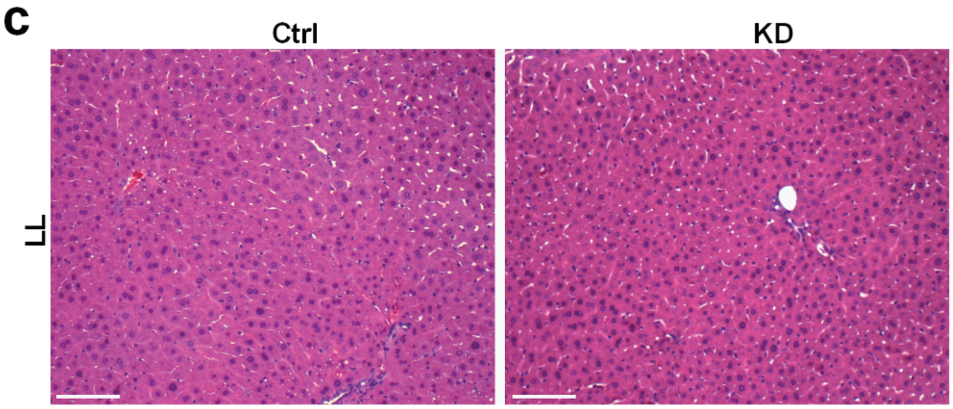

(a) mRNA (left panel) and protein (right panel) expression levels of MICU1, MICU2 and MCU in whole liver from Ctrl and KD mice. Bottom panel shows a representative immunoblot of MICU1, MICU2, MCU and GRB2 (as loading control). Relative mRNA levels are shown using β-Actin as an endogenous control and normalized to the Ctrl liver for each gene (n=8-12). Quantification of protein levels of MICU1, MICU2, MCU normalized to GRB2 in liver lysates from Ctrl and KD mice (n=6-8). (**b)** mRNA (right panel) and protein (left panel) expression levels of MICU1 from Ctrl and KD mice in whole tissue extracts from liver, spleen, lungs, kidney and heart respectively. Bottom panel shows a representative immunoblot of MICU1 and Vinculin (as a loading control) in liver (10μg), spleen (50μg), lung (50μg), heart (50μg), kidney (50μg) and blood (80μg) from Ctrl and KD mice. The relative mRNA levels are reported using β-Actin as an endogenous control and normalized to the Ctrl tissue for each organ (n=5). Quantification of protein levels of MICU1 normalized to Vinculin in respective tissue lysates from Ctrl and KD mice (n=3-5). **(c)** Representative images of H&E stained sections from Ctrl and KD liver. Scale bars: 50μm. Values are reported as mean ± SEM, *p<0.05, **p<0.005, ***p<0.0005 via two-way ANOVA followed by Tukey's multiple comparison test.

Supplementary Figure 4.

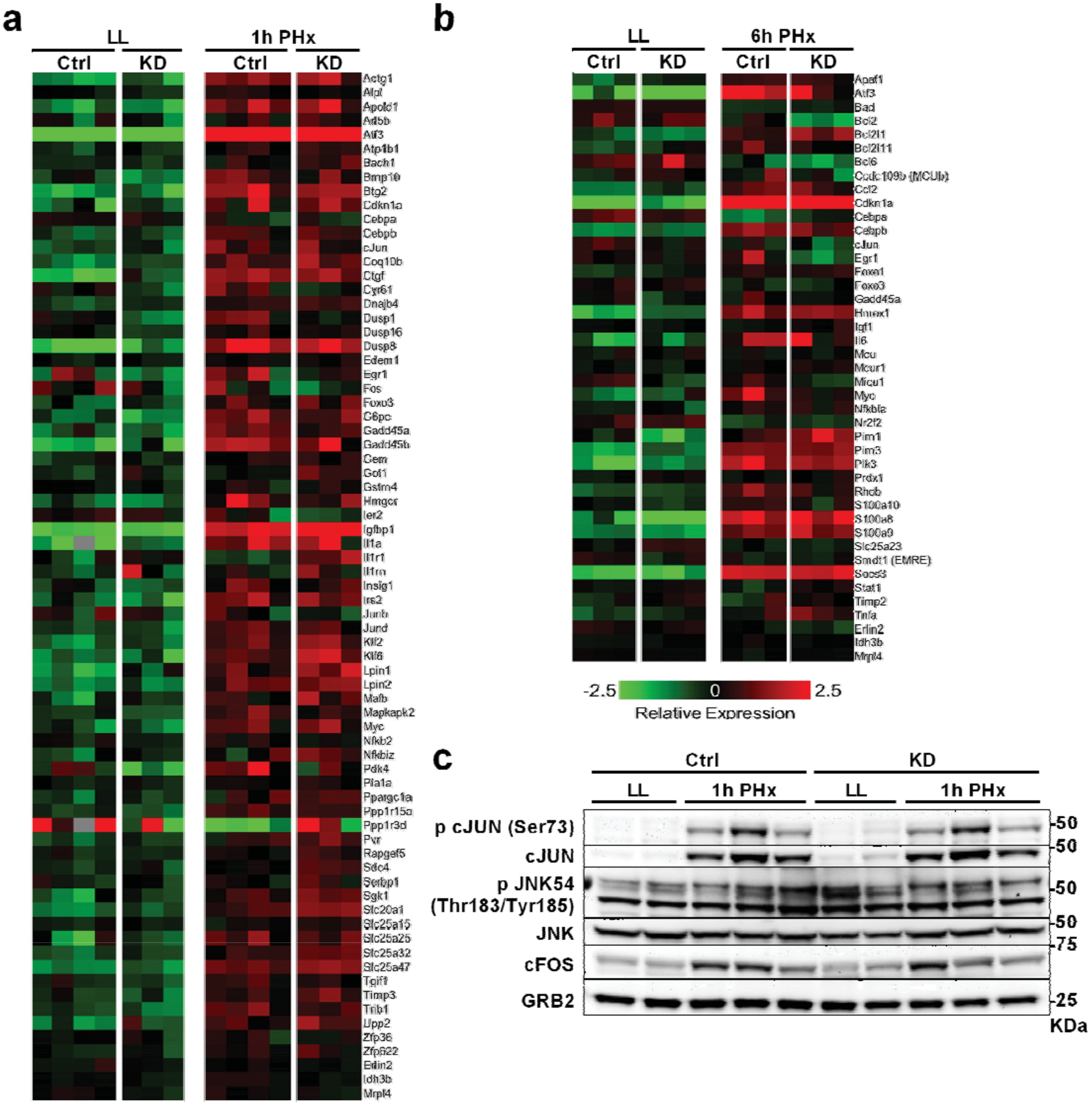

Supplementary Figure 4. (Cont'd)

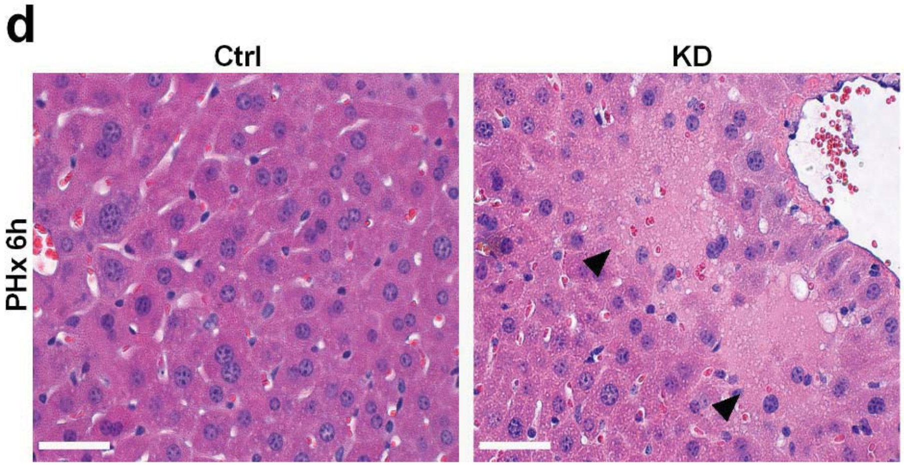

(a, b) Effects of MICU1 KD on the expression levels of select immediate-early signaling and apoptosis-related genes in liver before (LL) and at 1h (a) and 6h (b) after PHx. Expression was measured by high-throughput qPCR in tissue from Ctrl and KD mice and is presented as the mean-centered $-\Delta C_t$. **(c)** Representative immunoblotting of total and phosphorylated levels of cJUN (Ser73) and JNK54 (Thr183/Tyr185) and total level of cFOS in Ctrl and KD liver homogenates before (LL) and after 1h PHx. **(d)** Representative high magnification (63X) images of H&E stained sections from Ctrl and KD liver 6h post-PHx. Magnification displays necrotic area in KD liver 6 hours after PHx. Scale bars: 50 μm.

Supplementary Figure 5.

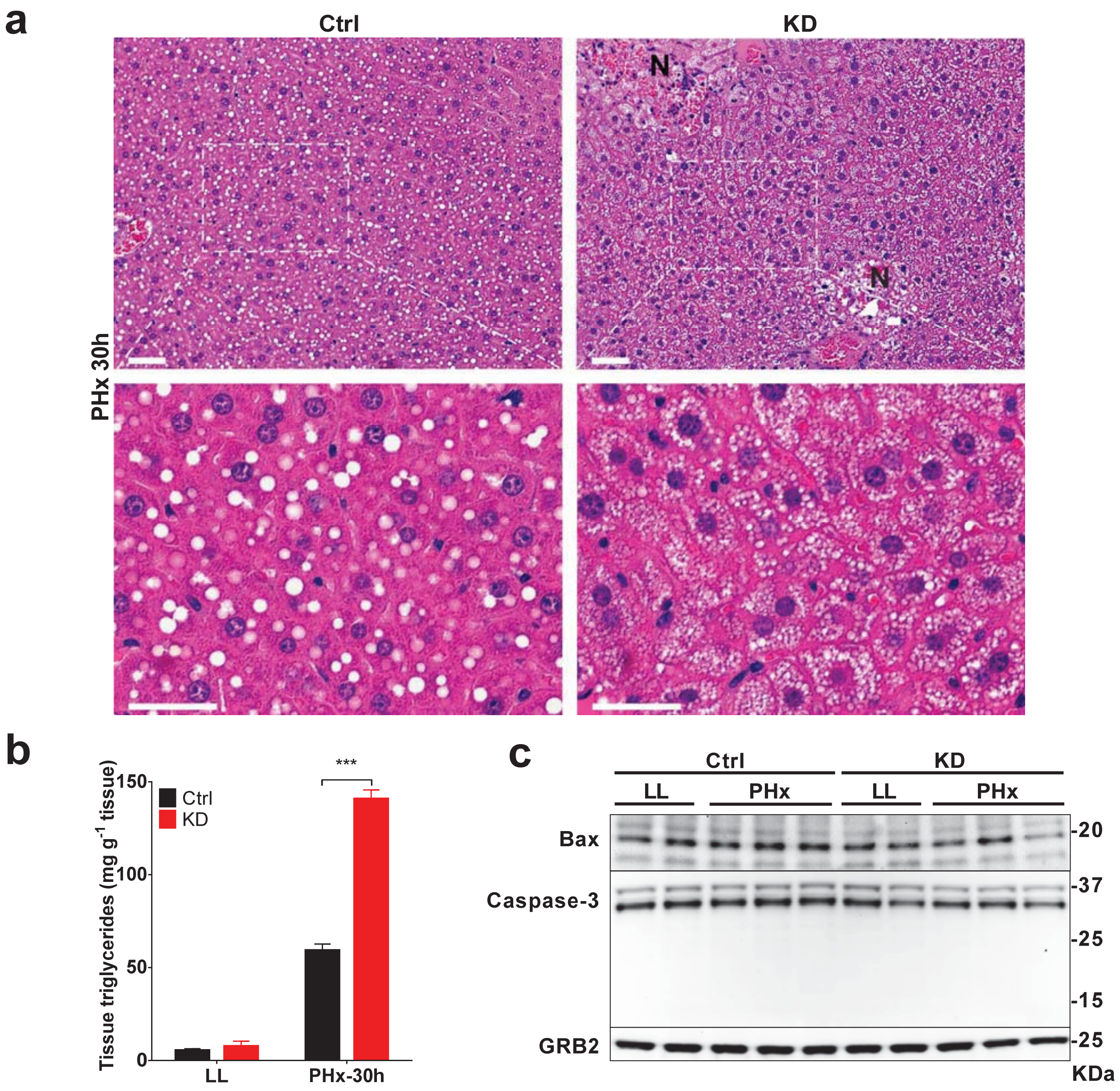

Supplementary Figure 5. (Cont'd)

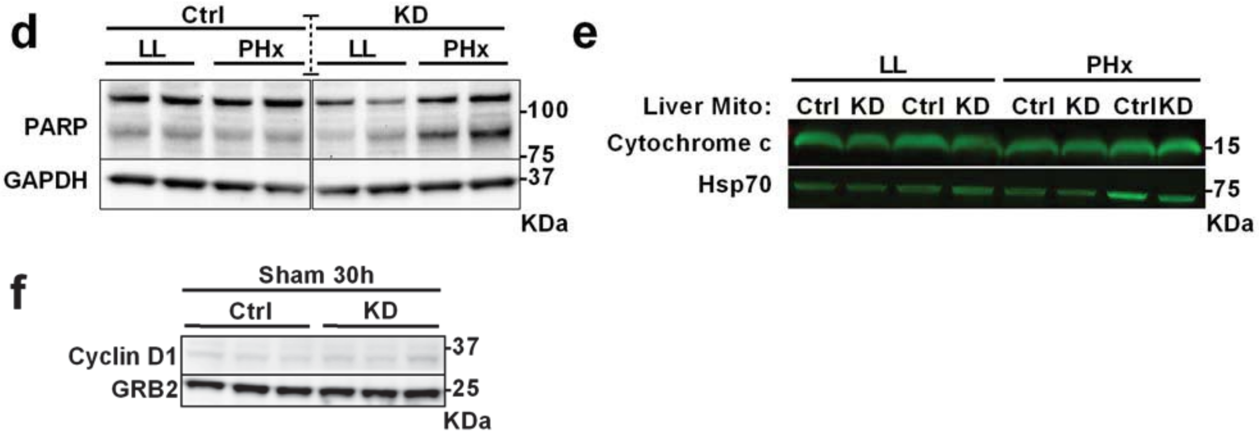

(a) Representative low (20x) and high magnification (63X) images of H&E stained sections from Ctrl and KD liver showing steatosis 30h post-PHx. Scale bars: 50 µm. **(b).** Tissue level of triglycerides in Ctrl and KD mice after 30h of PHx. (mean ± SEM, n= 3/group, ***p<0.0005 via two-way ANOVA followed by Tukey's multiple comparison test). **(c)** Representative immunoblotting of Bax, Caspase-3 and GRB2 (as a loading control) in Ctrl and KD liver homogenates before (LL) and after 30h PHx. **(d)** Representative immunoblotting of PARP and GAPDH (as loading control) in ctrl and KD liver homogenates before (LL) and after 30h PHx (images were taken from the same membrane as shown in supplementary figure 7) **(e)** Representative immunoblotting of Cytochrome c and Hsp70 (as loading control) in liver mitochondria isolated from ctrl and KD liver Pre-PHx from the left lateral lobe (LL) and 30h post-PHx from the right lobe. **(f)** Cyclin D1 expression assessed by immunoblotting in Ctrl and KD liver lysates 30h after sham surgery. n=3/group.

Supplementary Figure 6.

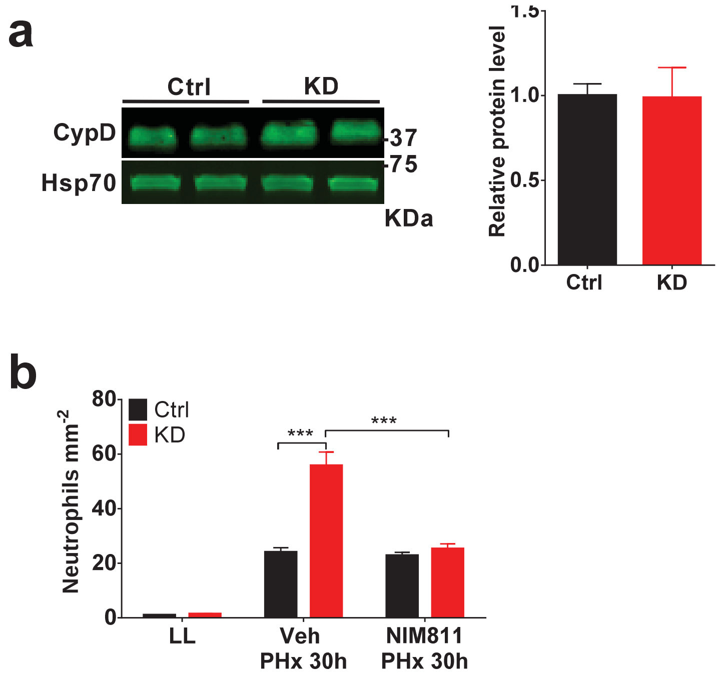

(a) Representative immunoblotting of CypD and Hsp70 (as a loading control) in Ctrl and KD hepatocytes. Relative protein level is displayed in the bar graph. CypD levels were normalized to Hsp70, and then normalized to Ctrl hepatocytes (mean ± SEM, n=4). **(b).** Neutrophils count using Ly-6g immunostaining in sections of Ctrl and KD liver 30h post-PHx with and without NIM treatment (mean ± SEM, n=4-5/group, ***$p<0.0005$, two-way ANOVA followed by Tukey's multiple comparison test.).

Supplementary Figure 7. Uncropped Immunoblot images

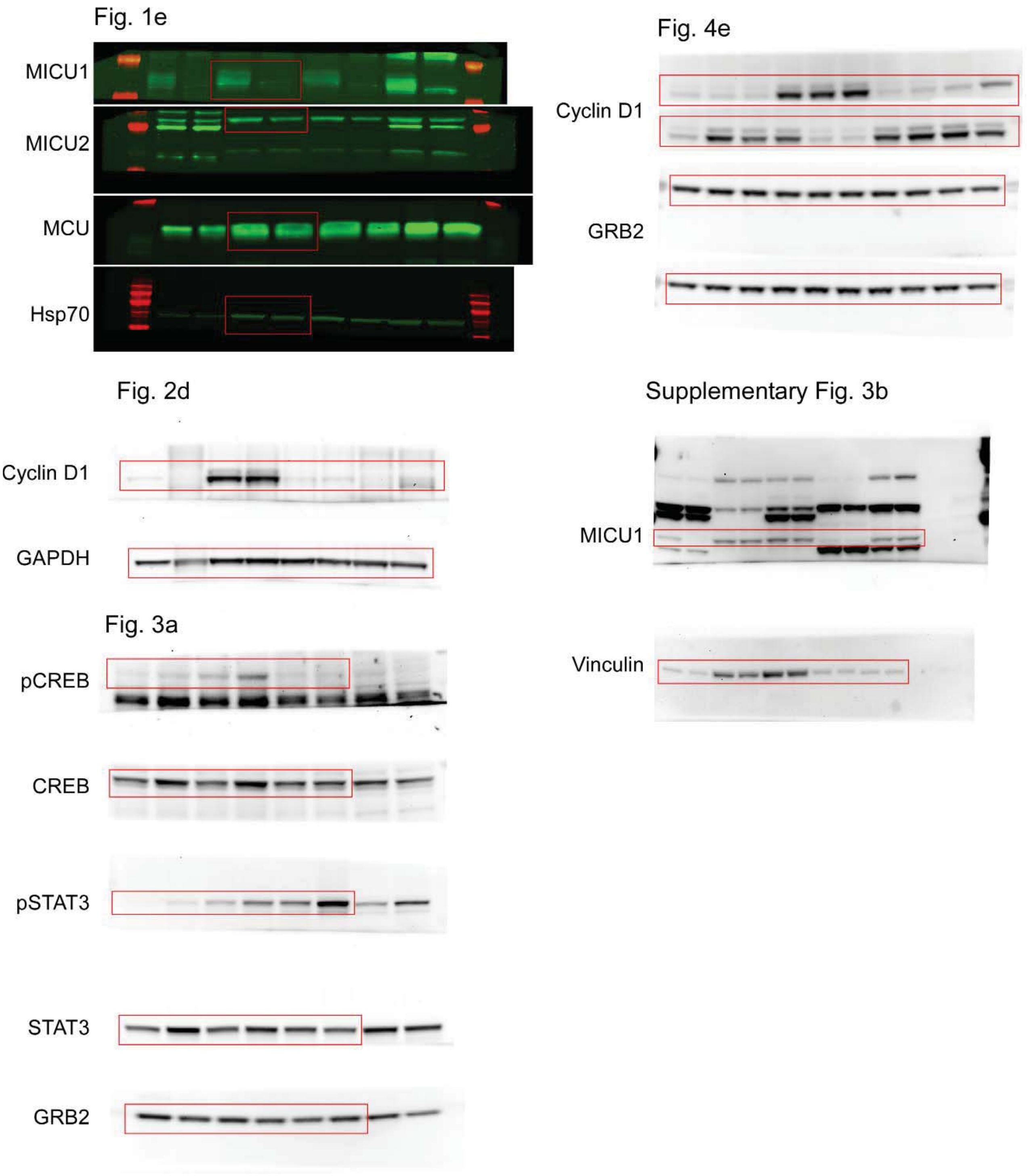

Supplementary Figure 7. Uncropped Immunoblot images (Cont'd)

Supplementary Fig. 4c

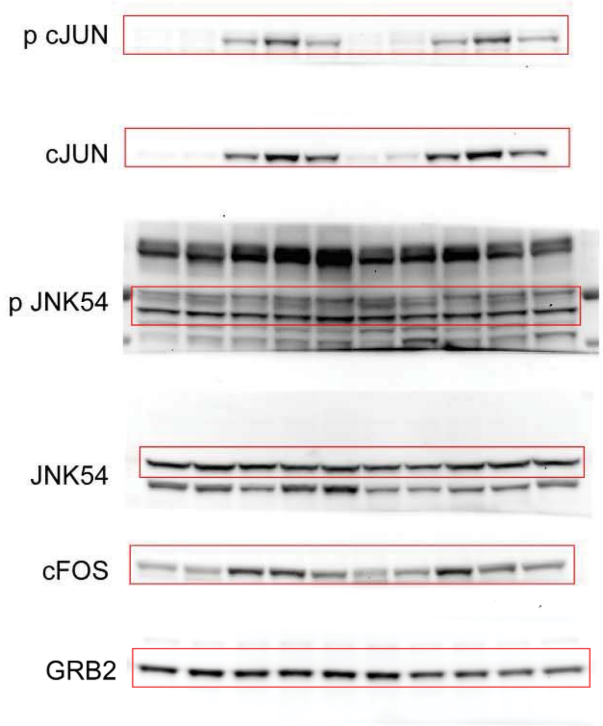

Supplementary Fig. 5c

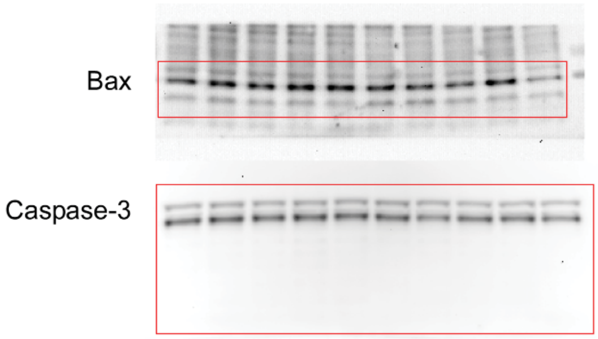

Supplementary Fig. 5d

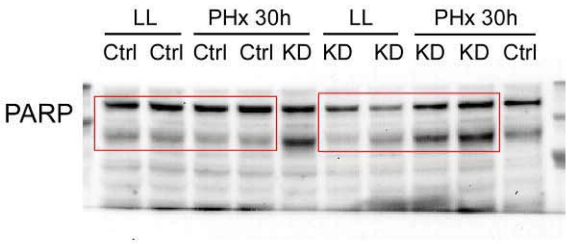

Supplementary Fig. 5e

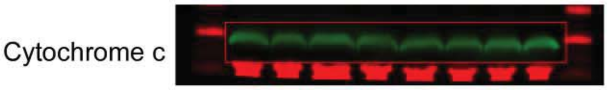

Supplementary Fig. 6a

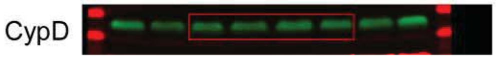

Supplementary Table 1: Body weight, blood glucose and gonadal fat % from fed Ctrl and KD mice

	Ctrl	KD
**Body weight(g)**	26.2 ± 0.5	27.3 ± 0.5
**Blood glucose (mg/dL)**	159.5 ± 4.7	155.0 ± 11.0
**% Gonadal white adipose tissue**	1.3 ± 0.2	1.4 ± 0.3
